# Recurrent Diverticulitis Complicated by Colovesicular Fistula and Multiloculated Abscesses Formation: A Bounce-Back Case Emphasizing Diagnostic Pitfalls and Multidisciplinary Management

**DOI:** 10.7759/cureus.101932

**Published:** 2026-01-20

**Authors:** Brian Lawton, Rene Ramirez

**Affiliations:** 1 Department of Emergency Medicine, University of California San Francisco Fresno, Fresno, USA

**Keywords:** antibiotic resistance, atypical presentation, bladder wall abscess, colovesicular fistula, diverticulitis, prostatic abscess

## Abstract

Diverticulitis complications, though rare, carry high morbidity. We present a 64-year-old male patient with multiple comorbidities who returned to the emergency department (ED) 27 days following hospital admission for acute diverticulitis with recurrence of the same, now complicated by colovesicular fistula, prostatitis, and multiloculated abscesses despite initial antibiotic therapy. Initial exam findings of tachycardia, and an appreciated boggy and severely tender prostate prompted timely/aggressive diagnostics and treatment. Computed tomography (CT) with intravenous (IV) contrast revealed a 6.6 cm prostatic abscess, 4.6 cm bladder abscess, and colovesicular fistula ultimately requiring Interventional Radiology drainage, broad-spectrum antibiotics, and urosurgical intervention. Cultures grew polymicrobial flora, including *Klebsiella* and *Pseudomonas*. This case underscores the need for early imaging in diverticulitis with urinary symptoms, multidisciplinary coordination for complex abscesses, and antibiotic stewardship in bounce-back patients. Emergency physicians must consider fistulae in high-risk populations to prevent delayed diagnosis or disease complications. Obtaining CT imaging with IV contrast early in patients who present with known diverticulitis in combination with acute urinary symptoms is important.

## Introduction

Diverticulosis is a relatively common disease affecting more than half of the United States population by the age of 60 years [1]. Up to 15% of these individuals will go on to develop an acute episode of diverticulitis [2].

Acute diverticulitis can be subdivided into two main categories: uncomplicated and complicated. Uncomplicated diverticulitis has been defined as localized swelling of intestinal diverticula in isolation from complicating factors (eg, abscess, fistula, hemorrhage, obstruction, perforation). Complicated diverticulitis is differentiated from its uncomplicated counterpart by the presence of one or more of the aforementioned complicating factors [3].

With the incidence of diverticulitis seemingly increasing for the general population, and the roughly 12% rate of complications (most commonly phlegmon or abscess formation), it is imperative that emergency physicians always consider the worst-case scenario (eg, peritonitis, obstruction, and fistula formation) when evaluating and treating a patient with such a condition [4]. This is especially important when the symptoms of such complications can be vague, attributable to multiple etiologies, and/or seemingly unrelated. With this in mind, we present a case of an emergency department (ED) bounce back for acute diverticulitis complicated by acute colovesicular fistula, prostatitis, and multiple/complex abscess formations.

## Case presentation

A 64-year-old male patient with a history of asthma, coronary artery disease, type 2 diabetes, hypertension, and prior ischemic stroke initially presented to an outside hospital with complaints of abdominal pain for approximately three to four days. At the outside facility, he was ultimately diagnosed with uncomplicated acute diverticulitis via CT abdomen/pelvis with contrast and received treatment with four days of intravenous (IV) Unasyn (ampicillin/sulbactam). At the time of his discharge, he was documented to be afebrile, with significant improvement in his level of pain, and a plan to continue outpatient treatment with a 10-day course of ciprofloxacin (500 mg PO twice a day) and metronidazole (500 mg PO three times a day).

Ten days following his hospital discharge, he presented to our ED with a complaint of dysuria and worsening lower abdominal pressure for three days. At this presentation, he was tachycardic (heart rate (HR) 109 beats per minute) and tachypneic (respiratory rate (RR) 20 breaths per minute), but his vital signs were otherwise unremarkable. Notably, he reported nonadherence with the recommended outpatient therapy, citing gastrointestinal upset as his reasoning for discontinuing metronidazole use after only one to two days of complete treatment adherence. During his roughly three-hour ED stay, he received a broad physical examination (including genitourinary (GU) exam), urinalysis (showing isolated glycosuria >1,000), and tests for Gonorrheae/Chlamydia (both of which were negative). Unfortunately, no repeat CT imaging was obtained during this visit, nor was there documentation to explain the provider's thought process as to why this was determined to be unnecessary. He was given a dose of naproxen, prescribed Pyridium and naproxen for his dysuria, and instructed to follow up with his primary care doctor.

Seventeen days later, the patient returned to the ED, where the authors of this report first met him, now complaining of two to three days of worsening dysuria and malodorous urine. During our initial evaluation, he was noted to be tachycardic (HR 114 beats per minute), uncomfortable in appearance, without abdominal tenderness to palpation, rigidity, guarding, or rebound. Given the persistence and evolution of symptoms, a digital rectal exam (DRE) was performed to investigate the possibility of concomitant prostatitis. A diffusely boggy prostate with exquisite tenderness to palpation was appreciated, prompting immediate broad-spectrum antibiotics (intravenous piperacillin/tazobactam and vancomycin) and further investigation with diagnostic labs and CT.

Notable initial lab derangements included: leukocytosis (white blood cell count 15.8 10*3/uL (reference range, 4.0-11.0 10*3/uL) without bandemia or left shift), nonanion gap metabolic acidosis (bicarbonate 18 mmol/L (reference range, 22-28 mmol/L), lactic acid 3.2 mmol/L (reference range, 0.5-2.2 mmol/L), calculated anion gap 11), marked hyperglycemia (blood glucose 384 mg/dL (reference rangem 70-99 mg/dL)), and troponemia (troponin I 0.325 ng/mL (reference rangem </=0.040 ng/mL).

An electrocardiogram (EKG) was obtained, given his tachycardia and notable for a new right bundle branch block without evolving changes on repeat EKGs. Initially, new objective cardiac abnormalities were attributed to type 2 non-ST-segment elevation myocardial infarction (NSTEMI) (demand ischemia), though cardiology was consulted following uptrend in repeat two-hour troponin I (0.400 ng/mL).

**Figure 1:**
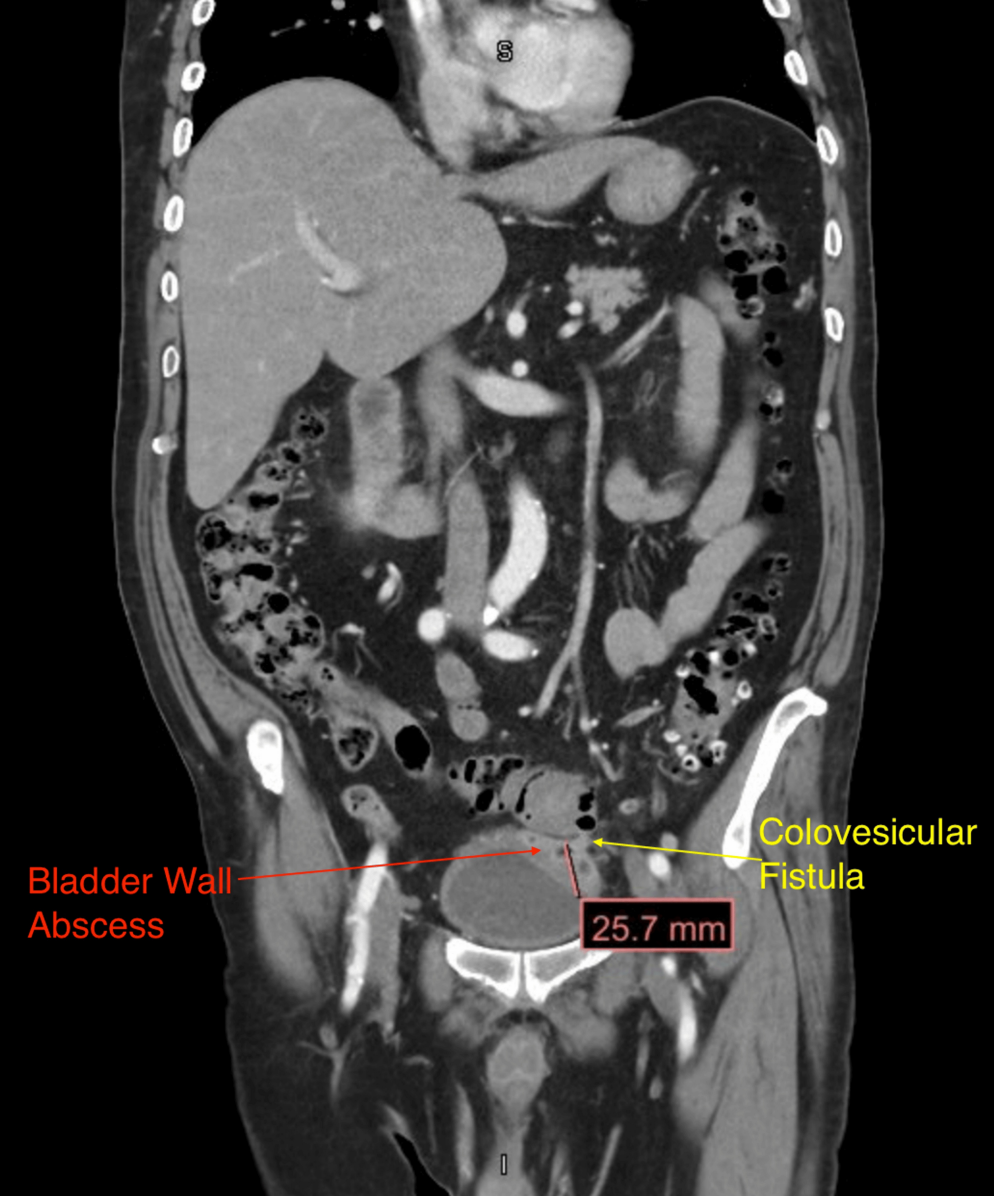
CT (coronal view) showing the colovesicular fistula and bladder abscess. Note the visible tract between the sigmoid colon and the bladder wall abscess with communication of air between the intestine and the fluid collection.

**Figure 2:**
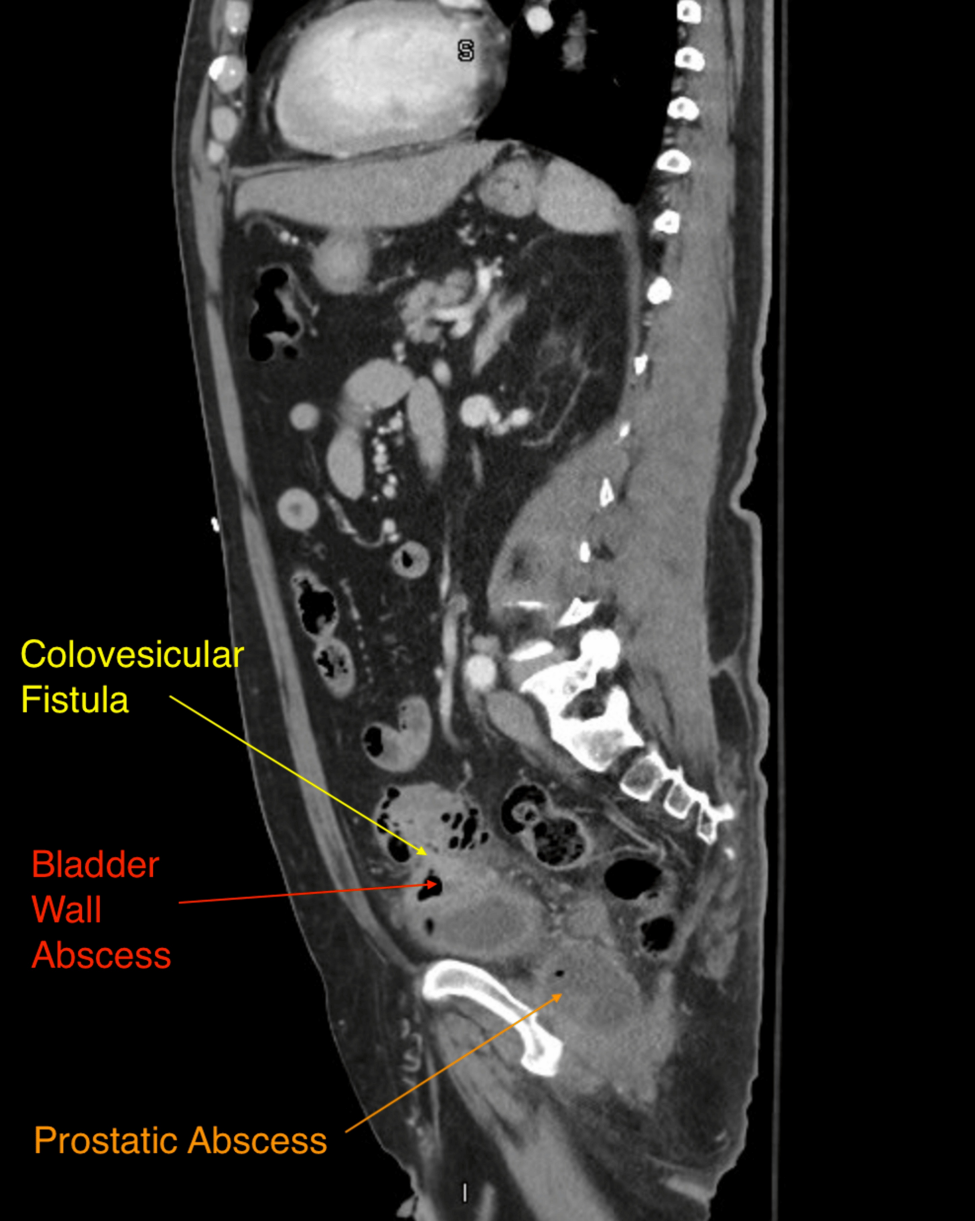
CT (sagittal view) showing colovesicular fistula, bladder abscess, and prostatic abscess

**Figure 3:**
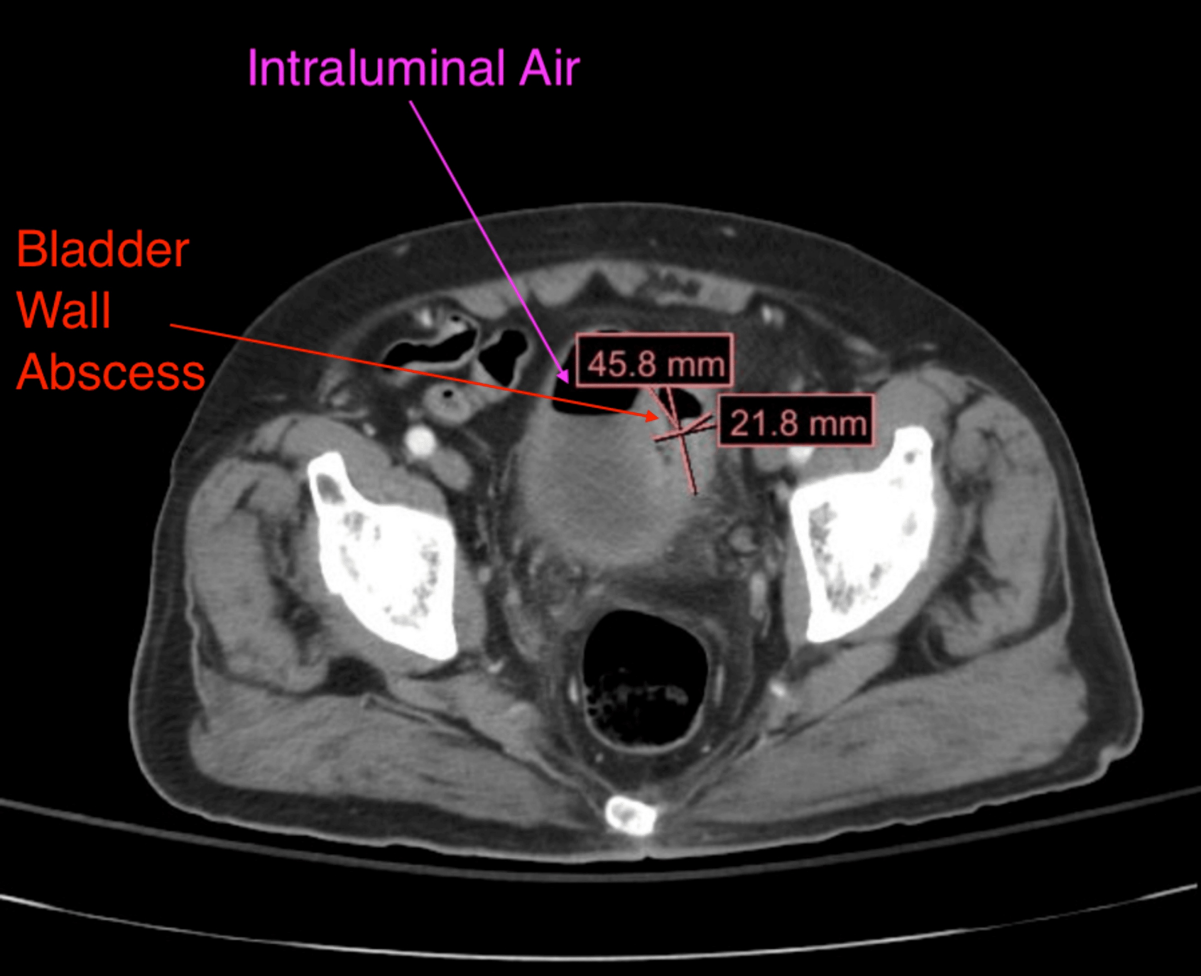
CT (axial view) showing a large bladder wall abscess and intraluminal air communicating from the large intestine

**Figure 4:**
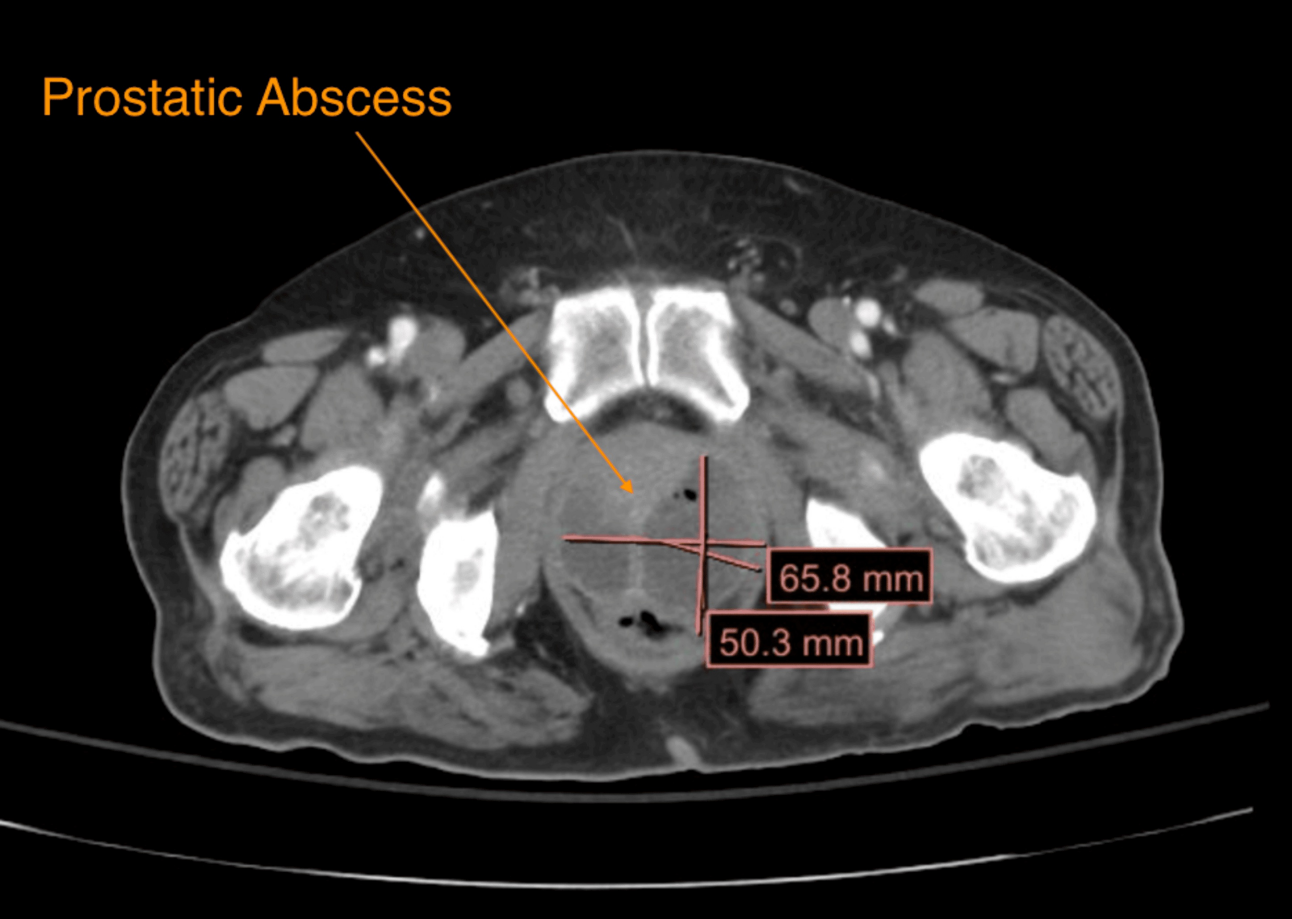
An axial view of the prostatic abscess. Note the size and bilobed/complexity of the fluid collection necessitating urologic consultation for operative drainage.

Most concerning of all, his CT (Figure 1) showed recurrence vs persistent acute sigmoid diverticulitis of the sigmoid colon, complicated by colovesicular fistula. There was an abscess in the left superior bladder dome measuring up to 4.6 cm (Figure 2). The colovesicular fistula appeared to be complicated by proctitis with a large bilobed air-containing abscess within the posterior prostate measuring up to 6.6 cm (Figures 3, 4). Inflammatory changes were seen throughout the perirectal fat and pelvis surrounding the diverticulitis, colovesicular fistula, and multifocal abscesses.

With this new information, in conjunction with his comorbidities, previous antibiotic nonadherence, and concern for possible necrotizing soft tissue infection, clindamycin was added to the patient’s antibiotic regimen for anti-toxin effects. His case was discussed with Colorectal Surgery and Urology before he was admitted to the Internal Medicine service for further inpatient workup and management.

While inpatient, he went on to receive an Interventional Radiology (IR)-placed temporary drain within the prostatic abscess; no attempt was made for drain placement within the bladder wall abscess, given IR’s concern for possible iatrogenic complications. Initial blood culture results became available within 24 hours of collection, showing growth of *Klebsiella pneumoniae*. Cultures sent from the IR placed drain grew multiple pathogens: *Klebsiella*, *Bacteroides*, *Streptococcus*, *Pseudomonas*, and *Candida *spps. Infectious Disease was thus involved in the patient’s care, and vancomycin was discontinued; fluconazole was added to his treatment regimen, given the complexity of the clinical scenario and concern for pathogenic yeast infection rather than colonization.

Unfortunately, the IR-placed prostatic drain was unable to fully evacuate the underlying abscess, ultimately necessitating the pursuit of transurethral resection of both the bladder and prostatic abscesses with Urology. Although Urology was successful in their resection of the prostatic abscess, they were unable to attempt to drainage/resection of the bladder wall abscess, given a documented inability to visualize structures necessary for safe intervention.

After continued medical stabilization, the patient was ultimately discharged from the hospital (on day 10 of inpatient course) with a peripherally inserted central catheter (PICC) and a plan for continued home IV Zosyn, oral fluconazole, and staged surgical correction of underlying structural abnormalities.

## Discussion

Acute diverticulitis is a common disease, and although fistula formation is a relatively rare complication, it remains the leading cause of colovesicular fistula formation today. Thus, it is our responsibility to always consider and investigate the possibility of colovesicular fistula formation in any/all patients with a diagnosis of acute diverticulitis who have concomitant urinary symptoms. Our case illustrates three critical diagnostic pitfalls: (i) atypical presentation/rare complications, (ii) imaging limitations, and (iii) antibiotic stewardship.

Our patient was diagnosed during his first visit with acute, uncomplicated diverticulitis. We would argue, on return to the ED at his subsequent visit, he should have received advanced imaging (eg, CT with IV contrast) to investigate for known complications of diverticulitis (especially in the setting of treatment nonadherence). As his urinalysis did not show any overt evidence of an infection, one may presume that this could be used as a screening tool to rule out the need for CT imaging. However, we would argue this is a falsehood and may lead providers to underdiagnose complicated diverticulitis as we present in this case. Remember, fistulas occur as a sequential complication and, for all intents and purposes, are the end result of a chronic inflammatory response. If our patient had received repeated CT imaging at the time of his first return visit to the ED, we suspect he likely would have been diagnosed with acute complicated diverticulitis, though the degree of his complication(s) may not have been as severe.

Although CT imaging with IV contrast is readily available in the ED, it is documented to be somewhat limited in its ability to accurately differentiate between the Hinchey classifications of acute diverticulitis [3]. Therefore, we would suggest that in cases of diagnostic uncertainty, or if there is a high enough clinical suspicion for complications of diverticulitis, pursuit of the gold standard diagnostic test (eg, CT abdomen/pelvis with oral contrast) should be undertaken [5].

Antibiotic stewardship is a mainstay of medical management in the modern era of medical practice. It is known that all pathogens can/will develop some degree of acquired antimicrobial resistance with each use. Patient factors (including, but not limited to, treatment nonadherence, immunodeficiency, health literacy, access to healthcare, etc.) further complicate things and highlight the importance of a methodical and tailored approach to clinical decision making and treatment recommendations [6]. Our patient clearly did not understand the importance of his outpatient medication regimen or his return precautions. As a result, these factors likely contributed to the complications highlighted in this case.

Colovesicular fistulae account for 65-80% of enterovesical fistulae and can be underrecognized in initial ED encounters due to nonspecific urinary symptoms [7,8]. Our patient’s dysuria and malodorous urine mimicked a urinary tract infection, delaying fistula identification. CT with oral/rectal contrast is the currently recommended first (and best) diagnostic modality of choice, with sensitivities approaching 90-100% [9]. However, given the undifferentiated nature of patients who present to the ED (and their multiple potential pathologies, which may preclude them from the pursuit of oral or rectal administration of contrast), in our experience, a CT abdomen/pelvis with IV contrast can be considered a safe and effective alternative in the initial ED diagnostic workup. Another alternative may be the use of transabdominal ultrasound, as there is some data to suggest equivalent sensitivities in identifying fistula formation in Crohn's disease [10,11]. However, extrapolation/application of this data to the diagnostic work-up in acute diverticulitis has yet to be proven, and there are a multitude of patient and provider factors that may limit the feasibility/reliability of ultrasonography.

The mainstay of treatment involves systemic antibiotics if signs/symptoms of an infection are present, as well as surgical correction of the underlying structural abnormalities, tailoring the surgical intervention to hospital capabilities, patient fitness for operative intervention, and projected surgical outcomes. Although complications can arise from acute diverticulitis despite appropriate treatment, nonadherence may contribute to the development of disease complications. As such, ED discharge plans must address medication tolerance and a patient’s health literacy, thus highlighting the importance of physician/patient communication. Current data suggest that there are a variety of social determinants of health that appear to play a major role in readmission rates [12]. First-line agents (e.g., ciprofloxacin/metronidazole) fail to cover resistant *Klebsiella* and *Pseudomonas* prevalent in nosocomial infections [13]. Our case supports the Infectious Diseases Society of America guidelines [14] recommending piperacillin-tazobactam for bounce-back cases. 

Prostatic abscesses >5 cm often require combined IR drainage and urologic resection, as percutaneous drainage alone fails in 60% of cases [15]. This patient’s 6.6 cm abscess necessitated transurethral debridement as the size and viscosity of the thick, fibrinous, and loculated fluid were not amenable to percutaneous drainage based on the opinions of our Urology team, reflecting standard of care treatment recommendations for complex pelvic abscesses [16]. Rising antibiotic resistance and cost-conscious care demand meticulous diagnostic evaluation and early specialist involvement. As ED volumes rise, leveraging imaging and specialist networks for high-risk diverticulitis cases becomes imperative to prevent morbidity [17].

## Conclusions

As healthcare providers, it is imperative that we adhere to a systematic approach when considering a broad differential diagnosis in the treatment of our patients. It is even more important when faced with atypical presentations or rare disease complications. With the modern evolution of antibiotic stewardship, our knowledge of disease pathophysiology, and treatment outcomes, we can exercise our privilege as trusted healthcare professionals to enact positive change in our communities and for our patients. All of these facets of medical care aim toward a desire to provide better, more equitable, and less costly care; though in tandem, require a higher level of scrutiny and clinical gestalt to make the appropriate treatment determination for our patients.

This case illustrates three critical lessons for clinical providers, especially in the emergency setting: (i) bounce-back mitigation, (ii) high-risk red flags, and (iii) resource utilization. In cases of uncomplicated diverticulitis, structured discharge protocols, such as pharmacist counseling and follow-up scheduling, may reduce readmission rates and the development of complications. Patient understanding, ability, and autonomy are imperative in the maintenance of treatment adherence. If there is any diagnostic uncertainty or if there is a high enough clinical suspicion for acute complications of acute diverticulitis, CT imaging of the abdomen/pelvis with enteral contrast administration should be pursued, as it remains the gold standard diagnostic imaging modality at this time. In cases of a patient with known acute diverticulitis returning for reevaluation, our case would suggest that providers strongly consider obtaining repeat CT imaging in all patients with new/acute urinary symptoms. Finally, in patients with acute complicated diverticulitis, early multidisciplinary management improves clinical outcomes and reduces healthcare costs/hospital length of stay.
